# Resistance Pattern of Carbapenem on Enterobacteriaceae

**DOI:** 10.31729/jnma.4006

**Published:** 2018-12-31

**Authors:** Khilasa Pokharel, Bishwa Raj Dawadi, Chandra Prakash Bhatt, Satish Gupte, Beena Jha

**Affiliations:** 1 Department of Microbiology, Kathmandu Medical College and Teaching Hospital, Sinamangal, Kathmandu, Nepal; 2 Department of Emergency Medicine, Grande International Hospital, Dhapasi, Kathmandu, Nepal

**Keywords:** *bacilli*, *carbapenem resistance*, *enterobacteriaceae*, *multidrug resistant*

## Abstract

**Introduction:**

Gram negative bacilli are the important causes of common clinical infections. Carbapenem resistant Enterobacteriaceae are considered as important public health threat and is classified as urgent by the Centers of Disease Control and Prevention because of their progressive geographic dissemination and limited therapeutic alternatives. This study was done to find out the resistance pattern of Carbapenem among Enterobacteriaceae.

**Methods:**

The descriptive cross-sectional study was carried out in Clinical Microbiology laboratory from February 2018 to May 2018 after ethical approval. Organism was identified on the basis of its microscopic observation by performing Gram's stain and by identification of morphology after its growth in culture media followed by its biochemical reactions. Antibiotic sensitivity test of isolated pathogens was done using Muller Hinton Agar by the standard disk diffusion technique of Kirby-Bauer method.

**Results:**

In our study, total 1055 sample belongs to the family Enterobacteriaceae. From the family Enterobactericeae, 348 (27%) of the bacilli were found to be Carbapenem resistant. Among which most common bacteria was Klebsiella pneumoniae followed by Escherichia coli. All strains of Car-bapenem resistant Enterobacteriaceae were sensitive to Colistin, Polymyxin B and Tigecycline.

**Conclusions:**

Among Enterobacteriaceae, around one-third of the bacterial isolates were Carbapenem resistant. However, to reduce drug resistance antimicrobial stewardship programme and proper infection control measures is required.

## INTRODUCTION

Gram negative bacteria are important cause of common clinical infections mainly in urinary tract infection, pneumonia, meningitis, septicemia etc and are also the normal intestinal flora.^1^ Antimicrobial resistance (AMR) is an emerging serious public health threat.^2^ Among clinically important Gram negative pathogens multidrug resistance is on rise.^3^

Although a lot of progress has been made for the treatment of the infection and relatively towards the progression in production of powerful antibiotics, the treatment of infectious disease is getting difficult. Mostly for the suspected or confirmed infections with multi drug resistant gram negative bacilli, including extended spectrum beta-lactamase (ESBL) producing Enterobac-teriaceae, Carbapenem therapy is reserved.^1^ In several regions around the world Carbapenem resistant Enterobacteriaceae (CRE) have emerged as a cause of nosocomial infection.^4^ Carbapenem resistance Enterobacte-riaceae are considered as important public health threat classified as urgent by the Centers of Disease Control and Prevention because of their progressive geographic dissemination and limited therapeutic alternatives.^4^

Hence, the aim of the study is to find out the resistance pattern of Carbapenem among Enterobacteriaceae.

## METHODS

This descriptive cross-sectional study was carried out in Clinical Microbiology Laboratory of Kathmandu Medical College and Teaching Hospital from the month of February 2018 to May 2018. Ethical approval was taken from Institutional Review Committee (IRC), Ref no 10/01/2018, KMCTH. Specimen like blood, urine, sputum, pus/wound swab, high vaginal swab, pleural fluid, ascitic fluid, cerebrospinal fluid, catheter tip, bile, stool, bronchoalveolar lavage, endotracheal tip, peritoneal fluid were collected following sterile precaution and specimen those were properly labelled was included. Those Clinical specimen that were not properly labelled and collected not following sterile precaution were left out. During the study period microbial data was obtained from the Clinical Microbiology Laboratory for identifying the prevalence of Carbapenem Resistance Enterobacteriaceae. Immediately after the collection of specimens, specimens were transferred to the Clinical Microbiology laboratory of KMCTH without delay for processing.

The sample size was calculated by using data from the study of (from the study done in Gian Sagar Medical College and Hospital, Rajpura, Punjab, India)^5^


$$\text{Sample size (n) =}{\text{Z}}^{2}\times \text{p}\times {\text{p/d}}^{2}=\text{approx}458$$

Where, z = value at Confidence interval at 99% p= prevalence of study i.e 50% q = 1-p

And, d= margin of error, 5%

Convenient sampling method was applied. All the positive cultures were examined microscopically by Gram's staining method. During observation; various factors including morphology of the organism (size, shape, arrangement), Gram's reaction of microbe, uniformity of the strain, pure or mixed form of organism, number of organism whether plenty, moderate or scanty were noted.

Identification of significant isolates were done by following standard microbiological techniques which involves morphological appearance of the colonies on MacConkey Agar which differentiate lactose-fermenter and non-lactose fermenter followed by Gram's staining reaction.^6^

Different biochemical tests were performed for the identification of the bacterial isolates. At first, pure culture were obtained from the primary culture and then, it was processed for biochemical tests. The biochemical media, employed was triple sugar iron agar (TSI) media, sulphide indole motility (SIM) media, Simmon's citrate media, Chirstensen's urea media.^6^

The antibiotic sensitivity tests of the pathogen isolated from clinical specimen against different antibiotic was done using Muller Hinton Agar (MHA) (HiMedia) by the standard disk diffusion technique of Kirby-Bauer method.^7^

A sterile cotton swab was dipped into broth and the swab rotated several times and pressed firmly on the inner side of the tube above the fluid level to remove excess inoculums from the swab. Then the dried surface of a Muller Hinton Agar plate was inoculated by streaking the swab over the entire agar surface three times, turning the plate at an angle of 60°C between streaking.^7,8^

After overnight incubation, the diameter of zone of inhibition (ZOI) of disk was measured (including the diameter of the disk) and recorded in millimeter.^7,8^

The descriptive statistical analysis was done using SPSS (Statistical Package for Social Services) 17.0.

## RESULTS

**Figure 1.**
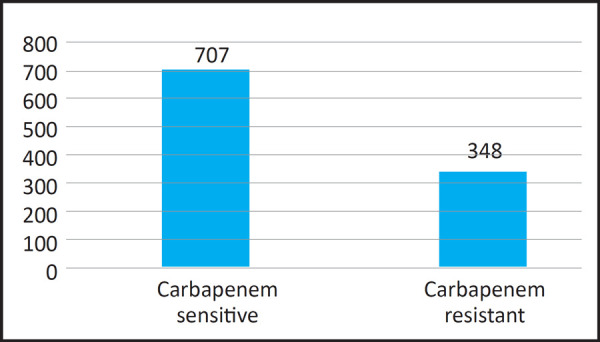
Carbapenem Sensitivity Pattern in Enterobacteriaceae.

Among 1055 Enterobacteriaceae, around one-third of the bacteria isolated were Carbapenem resistant (Figure 1).

**Figure 2.**
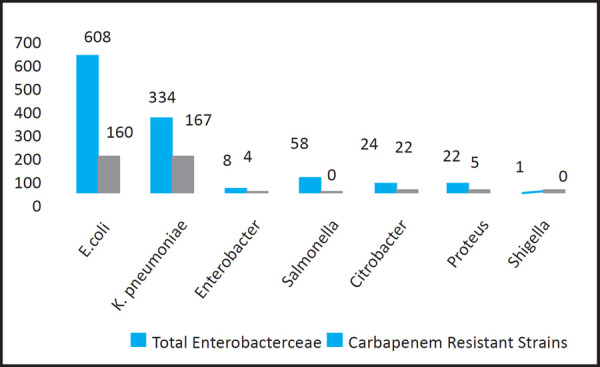
Carbapenem Resistance Among Enterobacteriaceae.

From those which belongs from Enterobacteriaceae, the most common bacteria isolated was Escherichia coli. However, Carbapenem resistance was mostly seen in Klebsiella pnuemoniae (Figure 2).

From the Enterobacteriaceae isolates, the most common bacteria seen were Klebsiella pneumoniae and Escherichia coli. The resistance pattern of Carbapenem was compared among these two bacteria to see bacterial resistance to antibiotic. Finally, it was seen that prevalence of resistance was seen more in K.pneumoniae 167 (50%) than E.coli 160 (26.3%) among the individual organisms (Table 1).

**Table 1:** Comparison of Carbapenem Resistance in E.coli and K.pnuemoniae.

	Carbapenem Resistance		Total	Chi-square value
	Yes n (%)	No n (%)		
E.coli	160 (26.3%)	448 (73.7%)	608	53.36
K.pneumoniae	167 (50%)	167 (50%)	334	

**Figure 3.**
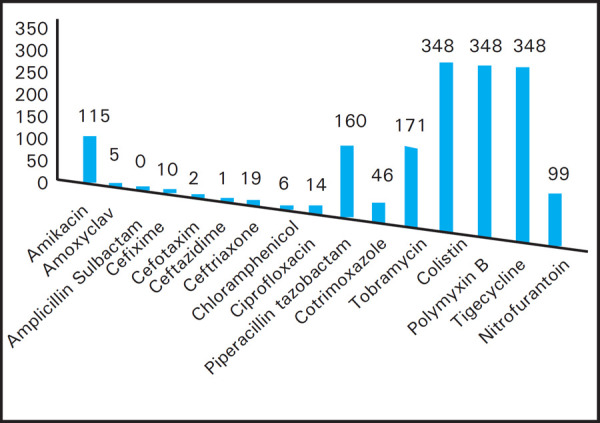
Antibiotic Resistance Pattern in Carbapenem Resistant Samples.

The Carbapenem resistant samples were also subjected to other antibiotic sensitivity test. All strains of Carbapenem resistant Enterobacteriaceae were seen sensitive to Colistin, Polymyxin B and Tigecycline. Similarly, all the Carbapenem resistant strain are found to be resistant to Ampicillin/Sulbactam. In addition to that, Amikacin, Piperacillin/Tazobactam and Tobramycin could also be used as good alternatives as almost half of the Carbapenem resistant Enterobacteriaceae were seen sensitive to them (Figure 3).

## DISCUSSION

Enterobacteriaceae is a group of bacilli that cause infection in community and health care setting. Resistance to broad- spectrum antibiotics is common among this group of bacilli.^8^

Our research on isolation of Carbapenem resistant Enterobacteriaceae from common clinical specimens obtained from Kathmandu Medical College and Teaching Hospital was processed from February 2018 to May 2018 and it showed alarming rate of drug resistance among Gram negative bacilli.

Out of 8781 samples that were processed, 17.95% were culture positive of which Gram negative bacteria were more in number as compared to that of Gram positive bacteria. As Anton Y,^9^ in their study mentioned that gram negative bacteria causes most of hospital acquired infections. They estimated that of all the hospital acquired infections, 30% of the infections were caused by Gram negative bacteria which are responsible for causing 70% of infection in hospital with Intensive care unit.^9^ Similar type of study was conducted by Ginawi et al,^10^ study shows among those patients who developed Nosocomial infections where 72.8% patients isolated pathogen were Gram negative bacilli. In our study out of 1266 gram negative isolates, 1055 bacilli belongs from the family Enterobacteriaceae.

Among positive blood culture isolates, 83 belong to family Enterobacteriaceae, from which the most common was Salmonella typhi 57, followed by Klebsiella pneumoniae 19, Escherichia coli 6 and Enterobacter sps 1, which is similar to the study conducted by Amatya et al,^11^ which shows out of 123 microbial growth in blood culture 78 were Salmonella typhi.

Total 658 gram negative bacilli were isolated from urine culture, of which 625 belongs from Enterobacteriaceae. The most predominant one was Escherichia coli 481, followed by Klebsiella pneumoniae 117, Proteus sps 13 and Enterobacter sps 4. This study is similar to the study conducted by Ayelign et al,^12^ which shows urine infection is mostly caused by Escherichia coli 54.88% followed by Klebsiella pneumoniae 4.88% and Proteus vulgaris 4.88%.

Except for blood and urine sample, we processed for other specimens in which, out of 686 positive samples, 492 were gram negative bacilli of which 347 belongs to Enterobacteriaceae. Of total 347 isolates the most predominant was Klebsiella pneumoniae (198), followed by Escherichia coli (121), Citrobacter sps (14), Enterobacter sps (3), Salmonella sps (1) and Shigella sps (1).

Out of 1055 Enterobactericeae, 348 were found to be resistant to Carbapenem, that means around one-third were isolated as Carbapenem resistant Enterobacteriaceae. From Enterobacteriaceae family, the most common bacteria isolated was Escherichia coli. However, Carbapenem resistance was mostly seen among Klebsiella pneumoniae.

In this present study, common organism isolated were Klebsiella pneumoniae and Escherichia coli. When the resistant pattern of Carbapenem was compared between these two organisms, the prevalence of resistance was seen in Klebsiella pneumoniae 167 (50%) as compared to Escherichia coli 160 (26%). This study is similar with the study of Bora et al,^13^ where out of 185 Klebsiella pneumoniae isolates 21.08% of isolates were suspected to be Carbapenemase producers on the basis of their reduced susceptibility to Meropenem and of 216 Escherichia coli isolates, total 41 isolates (18.98%) were suspected to be Carbapenemase producers.

Present study revealed, in urine culture 161 isolates of Enterobacteriaceae were resistant towards Carbapenem, but it shows its sensitivity towards Nitrofurantoin (NIT), similar kind of study has been conducted by Almugadam et al,^14^ where we can find 83.3% (10/12) sensitivity towards Nitrofurantoin among Carbapenem resistant Enterobacteriaceae.

When Carbapenem resistant samples were subjected to other antibiotic sensitivity test, all strains of Carbapenem resistant Enterobacteriaceae were found to be sensitive to Colistin, Polymyxin B and Tigecycline. Similar type of study was conducted by Lorenzoni et al,^15^ which show high level of sensitivity to Colistin (91.4%) among Klebsiella pneumoniae. Another study by Arnold et al,^16^ concluded that Klebsiella pneumonia Carbapenemase (KPC) producing bacteria have created challenges for clinicians due to highly drug resistance, resulting in delays in effective treatment so the effective antibiotics are limited to Polymyxin, Tigecyclines and occasionally aminoglycosides, which is similarto our study that shows predominance of KPC from Enterobacteriaceae showing 100% sensitivity towards Polymyxin B and Tigecycline.

Similarly, Amikacin, Piperacillin/Tazobactam and Tobramycin could be used as good alternatives as almost half of Carbapenem resistant Enterobacteriaceae were found sensitive to them. This study is similar to the study done by Almugadam et al^14^ and Lorenzoni et al^15^ which shows 83.3% (10/12) and increased rate 98.6% respectively, rate of sensitivity of Amikacin in Carbapenem resistant enterobacteriaceae.

## CONCLUSIONS

Among Enterobacteriaceae, around one-third of the bacteria isolated were Carbapenem resistant. Further, antimicrobial survey program and proper infection control tools are required for the control of pathogen. Extended studies are required to explore trend of antimicrobial resistance, to guide recommendation for antibiotic therapy for common infections.
